# Serum level of full-length connective tissue growth factor reflects liver fibrosis stage in patients with Fontan-associated liver disease

**DOI:** 10.1371/journal.pone.0296375

**Published:** 2024-01-02

**Authors:** Tomomi Kogiso, Kayo Takayanagi, Tsutomu Ishizuka, Motoyuki Otsuka, Kei Inai, Yuri Ogasawara, Kentaro Horiuchi, Makiko Taniai, Katsutoshi Tokushige

**Affiliations:** 1 Institute of Gastroenterology, Department of Internal Medicine, Tokyo Women’s Medical University, Tokyo, Japan; 2 Division of Transplant Immunology, Central Clinical Laboratories, Tokyo Women’s Medical University, Tokyo, Japan; 3 Department of Gastroenterology and Hepatology, Okayama University Graduate School of Medicine, Dentistry, and Pharmaceutical Sciences, Okayama, Japan; 4 Department of Pediatric Cardiology and Adult Congenital Cardiology, Tokyo Women’s Medical University, Tokyo, Japan; Osaka Rosai Hospital: Osaka Rosai Byoin, JAPAN

## Abstract

**Background:**

Chronic liver disease leads to liver fibrosis, and an accurate diagnosis of the fibrosis stage is crucial for medical management. Connective tissue growth factor (CTGF) is produced by endothelial cells and platelets and plays a central role in inducing fibrosis in various organs. In the present study, we tested the validity of measuring the serum levels of two types of CTGF to estimate the biopsy-confirmed liver fibrosis stage.

**Methods:**

We used two detection antibodies targeting the N- and C-terminal of CTGF to measure the serum levels of two forms of CTGF consisting of its full length and its N-terminal fragment. We analyzed the level of CTGF (via enzyme-linked immunosorbent assay) and the liver fibrosis stage in 38 patients with Fontan-associated liver disease (FALD) (26 cases of which were diagnosed pathologically). Correlations were determined by multivariate analysis and the area under the receiver operating characteristic curve. The 65 patients with nonalcoholic fatty liver disease (NAFLD) were included as a disease control group for examination.

**Results:**

Full-length CTGF was significantly inversely correlated with liver fibrosis in patients with FALD. Although the platelet count was also associated with the liver fibrosis stage, full-length CTGF was more closely correlated with the fibrosis stage. Furthermore, the level of full-length CTGF was inversely associated with high central venous pressure. Conversely, the serum level of CTGF was not correlated with the fibrosis stage in NAFLD.

**Conclusion:**

The serum level of full-length CTGF may be useful for estimating the liver fibrosis stage in patients with FALD.

## Introduction

Connective tissue growth factor (CTGF), a cysteine-rich heparin-binding protein, is a multifunctional growth factor involved in the progression of fibrosis in various organs including the lungs, bowel, kidneys, and liver [1–4]. Several gene-modified animal studies have clearly demonstrated crucial roles of CTGF in the development of liver fibrosis [5, 6]. Full-length CTGF consists of four conserved domains: insulin-like growth factor-binding protein domain (module 1), von Willebrand factor type C domain (module 2), thrombospondin type I repeat domain (module 3), and C-terminal cysteine-knot I repeat domain (module 4) [7, 8]. CTGF exists in two types *in vivo*: full-length CTGF, which is mainly produced by endothelial cells, and the N-terminal CTGF fragment consisting of modules 1 and 2, which is produced through protease cleavage between modules 2 and 3 of full-length CTGF. Although the serum level of CTGF may be correlated with the liver fibrosis stage [9, 10], most conventional methods of determining the CTGF level use an antibody against module 2 of CTGF. However, this measures the total CTGF level, which is the sum of the full-length CTGF level and the N-terminal fragment level. The significance of measuring full-length CTGF and the N-terminal fragment separately [11] is largely unknown.

Noninvasive estimation of the liver fibrosis stage in patients with chronic liver disease is clinically important because patient management and outcomes differ according to the stage of fibrosis [12, 13]. In Fontan-associated liver disease (FALD), noninvasive estimation of liver fibrosis is difficult using conventional clinical parameters [14–16]. In patients with FALD, the platelet count [17] and the Model for End-stage Liver Disease (MELD) score (particularly in patients treated with warfarin; therefore, the MELD-XI score [18], which excludes the international normalized ratio) are reportedly associated with disease development. In contrast, nonalcoholic fatty liver disease (NAFLD), the Japanese practice guideline of NAFLD/nonalcoholic steatohepatitis proposed that the platelet count and fibrosis-4 (FIB-4) index are reliable fibrosis markers in patients with NAFLD [19, 20]. However, the correlation between these markers and the liver fibrosis stage remains unknown. Therefore, novel strategies to estimate the fibrosis stage in patients with these diseases are in high demand [21].

In this study, we measured the serum level of full-length CTGF using an antibody against module 4 of CTGF to test the validity of measuring the levels of two types of CTGF (full-length CTGF and N-terminal fragment) to estimate the biopsy-confirmed liver fibrosis stage in patients with FALD and NAFLD.

## Materials and methods

### Patient information

This observational single-center study was conducted at Tokyo Women’s Medical University Hospital (April 19, 2016- April 18, 2022). FALD was diagnosed based on liver enzyme abnormalities, liver imaging using ultrasonography and computed tomography, and/or liver pathology. Ultimately, 38 patients with FALD were enrolled in this study. Liver biopsy was performed in 26 patients with FALD to examine the fibrosis stage, and the CTGF level was examined. Patient information and clinical laboratory data were collected at the time of biopsy. The homeostatic model assessment for insulin resistance was calculated from the glucose and insulin levels to quantify insulin resistance and β-cell function [22]. For patients with FALD, we collected data regarding cardiac disease, age at Fontan operation, and age at diagnosis of either FALD or hepatocellular carcinoma. CTGF was measured at the time of an outpatient visit. Twenty-one patients were treated with warfarin, and their MELD-XI score was used to evaluate liver function [23].

As the control, we also examined the 65 patients with NAFLD at 74 time points including 8 patients who underwent sequential biopsy. NAFLD was diagnosed according to evidence-based clinical practice guidelines [19, 20]. We rechecked the diagnoses of all patients and excluded those that did not meet the diagnostic criteria. The pathological stages of NAFLD were evaluated in accordance with an established classification system [24].

### Serum samples

Whole blood samples were obtained from peripheral vessels and centrifuged to collect serum specimens, which were stored at –20°C until assay performance. In patients with FALD, 38 samples were obtained. Samples from 26 patients were collected during liver biopsy or surgical treatment of liver tumors, and 1 sample was obtained after the fibrosis status had been determined by autopsy. The other samples were collected during a recent visit to our hospital. Among the patients from whom samples were collected, five were suspected of having mild liver fibrosis and seven were suspected of having advanced liver fibrosis based on clinical data and imaging studies. In patients with NAFLD, samples were collected at 74 time points of the liver biopsy, including 8 patients who underwent sequential biopsy. Enzyme-linked immunosorbent assay (ELISA) for the full-length CTGF level and total CTGF level (full-length CTGF level + N-terminal CTGF fragment level) was performed using two ELISA kits (FUJIFILM Wako Pure Chemical Corporation, Osaka, Japan). In both kits, an antibody against module 1 of CTGF was used to detect CTGF. To measure the total CTGF level using a conventional ELISA kit, an antibody against module 2 of CTGF was used as the detection antibody. For specific measurement of the full-length CTGF level, an antibody against module 4 was used as the detection antibody in another ELISA kit. The N-terminal CTGF fragment level was calculated by subtracting the full-length CTGF level from the total CTGF level. All ELISAs were performed according to the manufacturer’s instructions. Briefly, after the serum samples were appropriately diluted with reference to the detection range, a 50 μL sample was applied to each well of the 96-well ELISA kit for the capture of CTGF. The full-length and total CTGF levels were determined using the corresponding detection antibodies. All assays were performed in duplicate, and the mean values were calculated.

### Cardiac catheterization

Cardiac catheterization was performed by a pediatric cardiologist using a routine procedure, and the central venous pressure (CVP) was measured.

### Statistical analyses

Data are presented as the mean values of the results for each sample. The full-length CTGF level, total CTGF level (full-length CTGF level + N-terminal CTGF fragment level), and N-terminal CTGF fragment level were plotted in a scatter plot against the liver fibrosis stage and platelet count. Pearson’s correlation coefficients were used to evaluate the correlations between the bivariate data. A p value < 0.05 was considered statistically significant. The area under the receiver operating characteristic curve (AUC) was used to determine which factors fit with the liver fibrosis stage. Multivariate regression analysis for prediction of the fibrosis stage was performed using the following factors: platelet count, FIB-4 index, MELD-XI, total CTGF level, full-length CTGF level, and N-terminal CTGF level. The odds ratio and 95% confidence interval (CI) were assessed.

### Study approval

This study was conducted in accordance with the principles of the Declaration of Helsinki and the ethical guidelines of Tokyo Women’s Medical University Hospital (Tokyo, Japan). The Institutional Review Board of Tokyo Women’s Medical University Hospital approved the study protocol (approval numbers 3891 and 4989). Written informed consent was obtained from all participants or parents. In the cases with younger age, we obtained consent from parents or guardians. The datasets used in this study are available from the corresponding author upon reasonable request.

## Results

### CTGF level and fibrosis stage in patients with FALD

**Fig 1:**
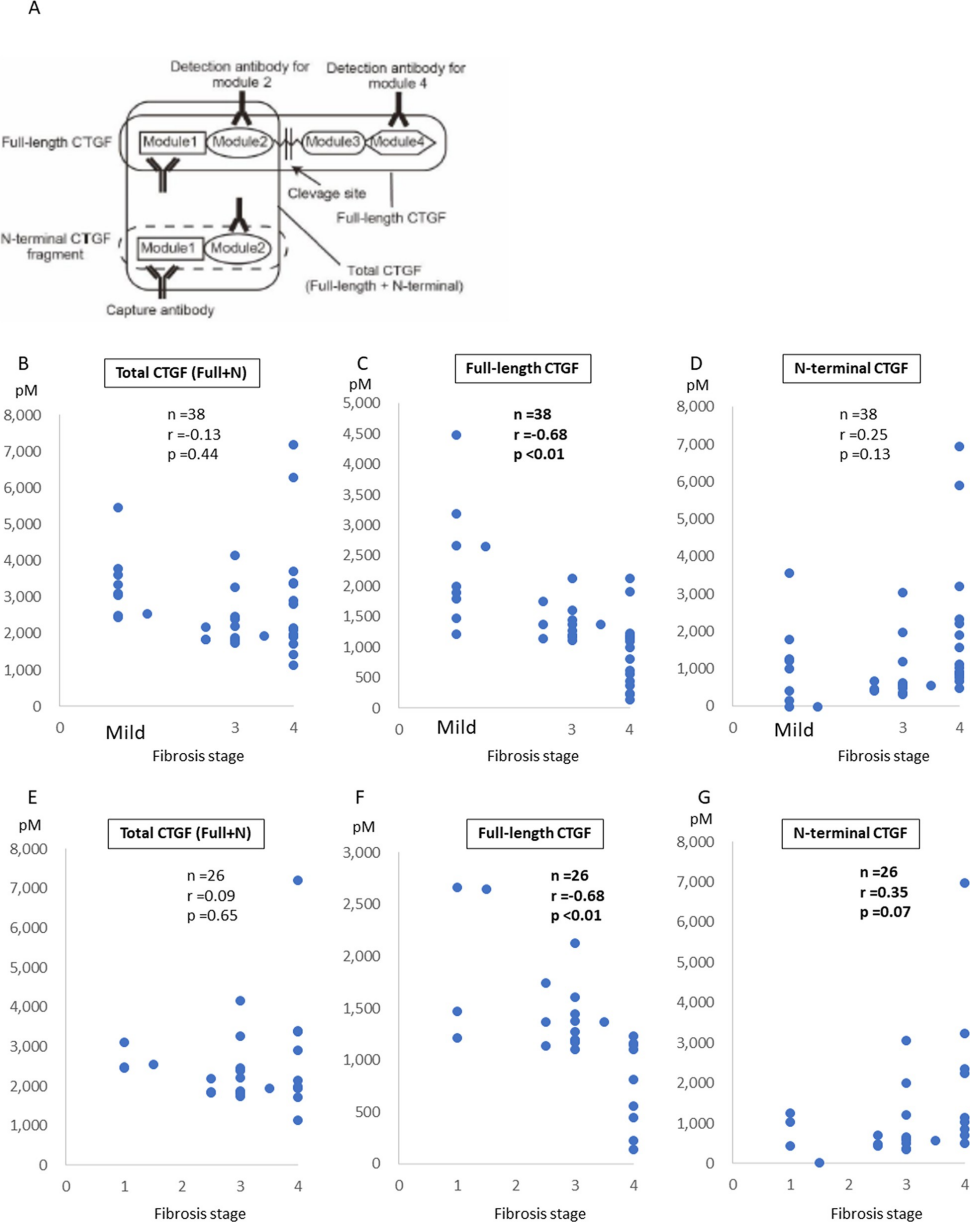
CTGF level in patients with FALD with different fibrosis stages. **(A)** Two sandwich ELISA assay systems were used. The full-length CTGF level was determined using the antibody against module 1 of CTGF as the capture antibody and the antibody against module 4 of CTGF as the detection antibody. The total CTGF level was determined using the antibody against module 2 of CTGF as the detection antibody, which detected the combined levels of N-terminal CTGF fragment and full-length CTGF. The N-terminal CTGF fragment level was determined by subtracting the full-length CTGF level from the total CTGF level. **(B, E)** The serum levels of total CTGF (full-length + N-terminal fragment), **(C, F)** full-length CTGF, and **(D, G)** N-terminal CTGF fragment were determined in 38 patients with FALD. The liver fibrosis stage was histologically confirmed in 26 patients. Mild fibrosis was assumed to be present in five patients based on their laboratory data and lack of liver cirrhosis characteristics in imaging. **(C)** The full-length CTGF level was significantly inversely correlated with the liver fibrosis stage (r = –0.68, p < 0.01). There were no statistically significant correlations between the fibrosis stage and the **(B)** total CTGF level or **(D)** N-terminal CTGF level. We verified histologically confirmed cases as well. **(F)** The full-length CTGF level was significantly inversely correlated with the liver fibrosis stage (r = –0.68, p < 0.01), and **(G)** the N-terminal CTGF level tended to be correlated; however, **(E)** the total CTGF level was not correlated. All data were determined in duplicate, and the average values are shown. CTGF, connective tissue growth factor; ELISA, enzyme-linked immunosorbent assay; FALD, Fontan-associated liver disease.

First, CTGF has been suggested to play critical biological roles in various fibrotic diseases, including liver fibrosis [10]; therefore, its serum level may be useful for diagnosing fibrosis progression [9]. To determine whether the serum level of CTGF is correlated with the progression of liver fibrosis in patients with FALD, we examined patients with FALD because no reliable fibrosis marker has been established for these patients and noninvasive fibrosis estimation methods are in high demand [15]. To determine the clinical significance of measuring the levels of two types of CTGF, we used two sandwich ELISA systems that measure the levels of full-length CTGF (detected by the antibody against module 4) and total CTGF (full-length CTGF + N-terminal CTGF fragment) (detected by the antibody against module 2). This enabled us to determine the full-length CTGF level and bioactive N-terminal CTGF level separately (Fig 1A). The serum level of CTGF was measured in 38 patients with FALD. The characteristics of these 38 patients are shown in Table 1. Fontan operation and diagnosis of FALD or hepatocellular carcinoma occurred at a median age of 13 (2–26) and 21 (7–43) years, respectively. Oral administration of anti-platelet agents was 26 cases (68.4%). Advanced liver fibrosis (fibrosis stage 3 and 4) was histologically confirmed in 19 (73.1%) of 26 patients. Liver biopsy was not performed in 12 patients, but 5 of these were assumed to have mild fibrosis based on their laboratory data and the lack of typical liver cirrhosis characteristics in their imaging examinations. The other seven were clinically assumed to have liver cirrhosis with typical findings, including ascites or esophageal varices. The median levels of total CTGF (full-length CTGF + N-terminal), full-length CTGF, and N-terminal CTGF were 2417.2, 1217.3, and 907.9 pM, respectively. Although the total CTGF level (r = –0.13, p = 0.44, Fig 1B) and N-terminal CTGF fragment level (r = 0.25, p = 0.13, Fig 1D) were not correlated with the liver fibrosis stage, the full-length CTGF level was significantly inversely correlated with the liver fibrosis stage (r = –0.68, p < 0.01, Fig 1C). Likewise, in patients with biopsy-confirmed fibrosis (n = 26), the total CTGF level was not correlated with the fibrosis stage (r = 0.09, p = 0.65, Fig 1E). By contrast, the full-length CTGF level was significantly inversely correlated with the liver fibrosis stage (r = –0.68, p < 0.01, Fig 1F), and the N-terminal CTGF fragment level tended to be associated with the fibrosis stage (r = 0.35, p = 0.07, Fig 1G). These results suggest that although the total CTGF level measured using conventional methods does not reflect the fibrosis stage in patients with FALD, specific measurement of the full-length CTGF level in serum may significantly reflect the fibrosis stage in patients with FALD. Oral administration of anti-platelet agents used in 26 (68.4%) of the cases. There was no significant difference observed in the total CTGF/ the full-length CTGF level/ the N-terminal CTGF fragment level between the patients with anti-platelet agents (2423.6, 1187.9, and 917.4) and those without (2443.5, 1223.2, and 689.0) (p = 0.85, 0.70, and 0.99, respectively). The use of anti-platelet agents did not show an association with CTGF levels in our study.

**Table 1:** Characteristics of patients with FALD.

	Total (n = 38)
Age at diagnosis of FALD or HCC (years)	21 (7–43)
Age at Fontan operation (years)	13 (2–26)
Male	16 (42.1)
**Cardiac disease**	
Single ventricle	7 (18.4)
Pulmonary atresia	6 (15.8)
Tricuspid valve insufficiency	8 (21.1)
Double-outlet right ventricle	14 (36.8)
Complete transposition of the great arteries	3 (7.9)
**Complications**	
Asplenia	7 (18.4)
Polysplenia	3 (7.9)
Visceral inversion	3 (7.9)
HCV antibody positivity	2 (5.3)
**Laboratory data**	
Albumin (g/dL)	4.7 (2.4–5.2)
Total bilirubin (mg/dL)	1.4 (0.5–5.2)
Aspartate aminotransferase (U/L)	24 (14–41)
Alanine transaminase (U/L)	20 (6–59)
Gamma-glutamyl transferase (U/L)	60 (22–235)
Platelet count (×10^4^/μL)	17.5 (6.2–50.1)
Prothrombin time (%)	96.0 (9.6–120.0)
FIB-4 index	1.47 (0.10–17.78)
BNP (pg/mL)	53.2 (14.3–577.0)
MELD-XI	12.53 (9.44–28.83)
**Oral administration of anti-platelet agents (%)**	26 (68.4%)
**ELISA assay of CTGF**	
Total CTGF (full-length CTGF + N-terminal) (pM)	2417.2 (1131.0–7185.8)
Full-length CTGF (pM)	1217.3 (134.8–4470.3)
N-terminal (pM)	907.9 (0.0–6963.4)
**Pathological findings**	
Fibrosis stage F3–4	19/26 (73.1)
**Cardiac catheterization**	
CVP (mmHg)	11 (4–20)

Data are presented as median (range) or n (%) unless otherwise indicated. BNP, brain natriuretic peptide; CTGF, connective tissue growth factor; CVP, central venous pressure; ELISA, enzyme-linked immunosorbent assay; FALD, Fontan-associated liver disease; MELD-XI, model for end-stage liver disease excluding the international normalized ratio; HCV, hepatitis C virus; HCC, hepatocellular carcinoma; FIB-4, fibrosis-4.

### Correlation between CTGF level and platelet count in patients with FALD

**Fig 2:**
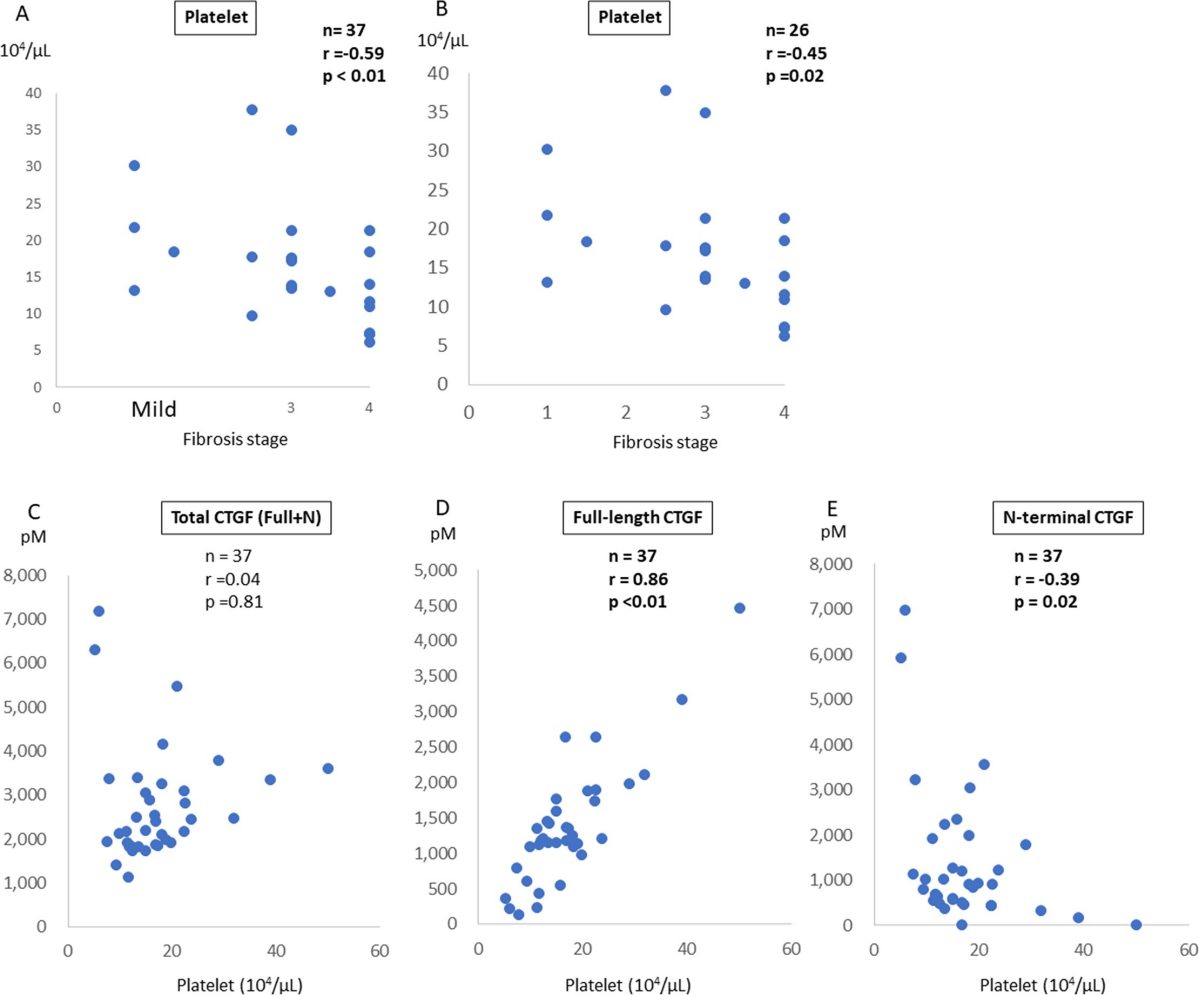
Correlation between platelet count and CTGF level in patients with FALD. **(A)** The correlation between the fibrosis stage and platelet count was determined in patients with FALD. **(B–D)** The correlation between the platelet counts and CTGF levels (total, full-length, and N-terminal) was also assessed. **(B)** The total CTGF level was not associated with the platelet count. **(C)** The full-length CTGF level was significantly correlated with the platelet count. **(D)** The N-terminal fragment level tended to be negatively correlated with the platelet count, although the correlation was not statistically significant. All CTGF levels were determined in duplicate, and the average values are shown. CTGF, connective tissue growth factor; FALD, Fontan-associated liver disease.

Although the full-length CTGF level is inversely correlated with the liver fibrosis stage in patients with FALD, it is possible that the changes in the full-length CTGF level may reflect the decrease in the platelet count due to progression of liver fibrosis because platelets contain full-length CTGF [7, 8]. In the present study, the platelet count was significantly correlated with progression of the liver fibrosis stage in patients with FALD (r = –0.59, p < 0.01, Fig 2A). The correlation was slightly reduced in biopsy-confirmed cases (r = –0.45, p = 0.02, Fig 2B). In addition, although the total CTGF level was not significantly correlated with the platelet count (r = 0.04, p = 0.81, Fig 2C), the full-length CTGF level (r = 0.86, p < 0.01, Fig 2D) and N-terminal CTGF fragment level (r = –0.39, p = 0.02, Fig 2E) were significantly correlated with the platelet count.

### Correlation between CTGF level and CVP in patients with FALD

**Fig 3:**
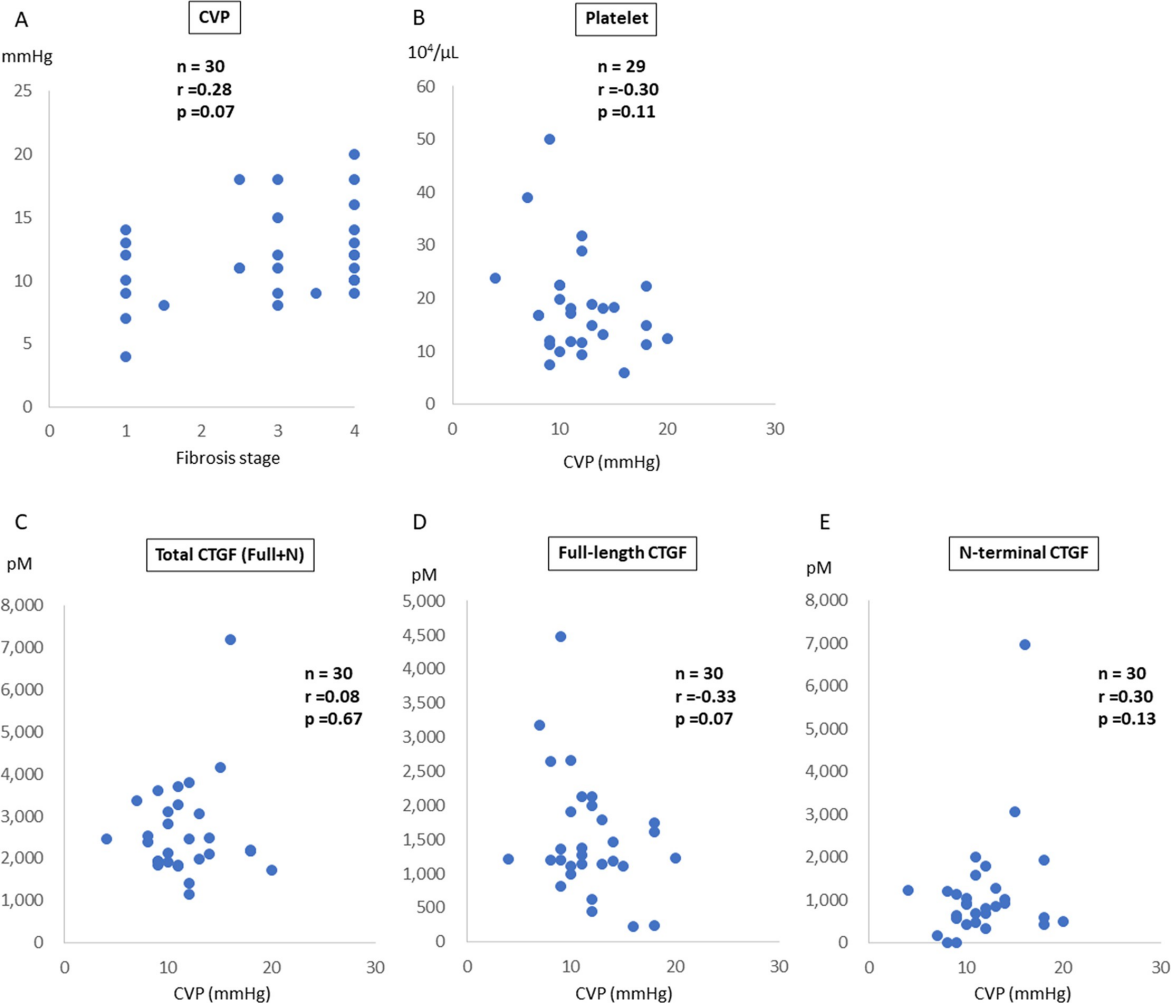
Correlation between CVP and CTGF level in patients with FALD. Correlation between CVP and **(A)** fibrosis stage, **(B)** platelet count, and **(C–E)** CTGF levels (total, full-length, and N-terminal). **(A)** CVP tended to be increased with fibrosis development. The **(B)** platelet count and **(C)** total CTGF level were not associated with CVP. **(D)** The full-length CTGF level tended to be negatively correlated with CVP. **(E)** The N-terminal fragment level tended to be correlated with CVP, although the correlation was not statistically significant. All CTGF levels were determined in duplicate, and the average values are shown. CTGF, connective tissue growth factor; CVP, central venous pressure; FALD, Fontan-associated liver disease.

CVP was measured via cardiac catheterization. The median CVP was 11 (4–20) mmHg. CVP tended to correlate with the fibrosis stage in patients with FALD (r = 0.28, p = 0.07, Fig 3A). However, there was no significant correlation with the platelet count (r = –0.30, p = 0.11, Fig 3B). Assessment of the CVP and CTGF level showed that the total CTGF level (r = 0.08, p = 0.67, Fig 3C) and N-terminal CTGF fragment level (r = 0.30, p = 0.13, Fig 3E) were not correlated with CVP; however, the full-length CTGF level was more likely to be correlated with CVP (r = –0.33, p = 0.07, Fig 3D).

### Accuracy of full-length CTGF level in predicting fibrosis stage in patients with FALD

We utilized a receiver operating characteristic curve to determine the accuracy of predicting the fibrosis stage in patients with FALD (S1 Fig). The AUC of the platelet count, FIB-4 index, and full-length CTGF was 0.744, 0.567, and 0.822, respectively. Full-length CTGF was a better predictor of fibrosis in patients with FALD. In the multivariate analysis, full-length CTGF was identified as the independent fibrotic marker of FALD (odds ratio, 0.997; 95% CI, 0.995–1.000, p = 0.04) among the platelet count, FIB-4 index, MELD-XI, total CTGF, full-length CTGF, and N-terminal CTGF. These results suggest that the full-length CTGF level may better reflect the liver fibrosis stage than other markers in patients with FALD, particularly patients with high CVP.

### CTGF level and fibrosis stage in patients with NAFLD

**Fig 4:**
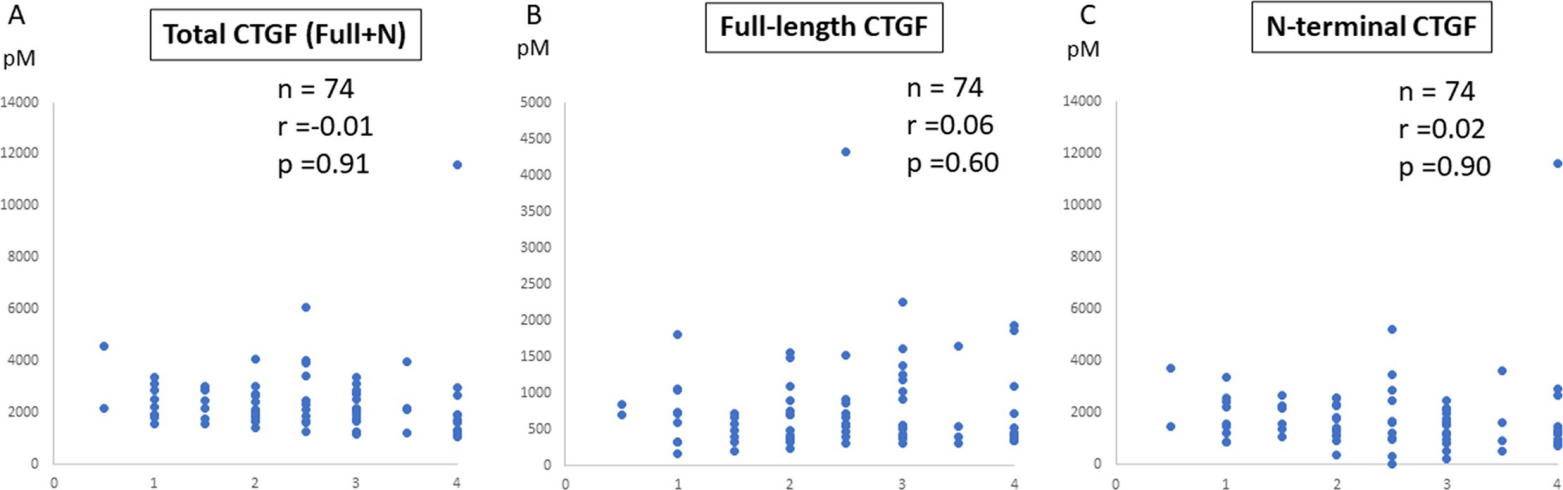
CTGF level in patients with NAFLD with different stages of biopsy-proven level fibrosis. The **(A)** total CTGF level (full-length + N-terminal fragment), **(B)** full-length CTGF level, and **(C)** N-terminal CTGF fragment level in serum from 14 patients with NAFLD with different fibrosis stages were measured. The N-terminal CTGF fragment level was determined by subtraction as described above. All data were obtained in duplicate, and the average values are shown. CTGF, connective tissue growth factor; NAFLD, nonalcoholic fatty liver disease.

Next, to assess the CTGF level if it was characteristic with FALD, we examined 65 patients with another etiology of liver fibrosis, NAFLD whose liver fibrosis stage had been confirmed by biopsy. The laboratory data at the time of biopsy are shown in Table 2. The patients’ median age at the time of biopsy was 51 (15–79) years. We evaluated data at 74 time points for these patients, including 8 patients who underwent sequential biopsy at 17 time points in total. The total CTGF (full-length CTGF + N-terminal), full-length CTGF, and N-terminal CTGF levels were 2121.9, 558.5, and 1397.1 pM, respectively. Pathologically, advanced fibrosis (fibrosis stage 3–4), advanced inflammation (inflammation grade 2–3), and advanced steatosis (steatosis grade 2–3) were observed in 31 (41.9%), 62 (83.8%), and 56 (75.7%) patients, respectively. We initially expected that because CTGF is a crucial factor in liver fibrosis and its serum level has been reported as a marker of fibrosis [9], its level should be associated with the progression of liver fibrosis in patients with NAFLD. However, measurement of CTGF in our study showed no significant correlation between the liver fibrosis stage and the total CTGF level (Fig 4A), full-length CTGF level (Fig 4B), or N-terminal fragment level (Fig 4C) in patients with NAFLD. The correlation of fibrosis stage and any CTGF level were not observed in NAFLD cases.

**Table 2:** Characteristics of patients with NAFLD.

**Variables**	NAFLD
	(n = 65 at 74 time points)
Age (years)	51 (15–79)
Male sex	36 (48.6)
BMI (kg/m^2^)	27.4 (17.6–43.3)
Dyslipidemia	46 (62.2)
Hypertension	38 (51.4)
Diabetes mellitus	38 (51.4)
**Laboratory data**	
ALB (g/dL)	4.4 (3.0–5.2)
T-BIL (mg/dL)	0.8 (0.4–4.9)
AST (U/L)	43 (13–153)
ALT (U/L)	61 (10–230)
GGT (U/L)	64 (13–452)
FBS (mg/dL)	105 (76–343)
Hemoglobin A1c (%)	6.0 (4.3–12.0)
IRI (μU/mL)	10.3 (1.6–43.8)
HOMA-IR	2.67 (0.34–19.03)
Triglycerides (mg/dL)	136 (41–593)
Total cholesterol (mg/dL)	184 (95–285)
Ferritin (ng/mL)	219 (13–774)
Platelet count (×10^4^/μL)	20.9 (6.1–96.0)
Prothrombin time (%)	90.5 (55.0–106.0)
Alpha-fetoprotein (ng/mL)	4.0 (2.0–8.0)
**ELISA of CTGF**	
Total CTGF (full-length CTGF + N-terminal) (pM)	2121.9 (1028.3–11606.8)
Full-length CTGF (pM)	558.5 (160.0–4327.3)
N-terminal (pM)	1397.1 (0.0–10523.4)
**Pathological findings**	
Fibrosis stage F3–4	31 (41.9)
Inflammation grade A2–3	62 (83.8)
Steatosis grade S2–3	56 (75.7)

Data are presented as median (range) or n (%) unless otherwise indicated. ALB, albumin; AST, aspartate aminotransferase; ALT, alanine transaminase; GGT, γ-glutamyl transferase; BMI, body mass index; CTGF, connective tissue growth factor; ELISA, enzyme-linked immunosorbent assay; FBS, fasting blood glucose; NAFLD, nonalcoholic fatty liver disease; HOMA-IR, homeostatic model assessment of insulin resistance; IRI, immunoreactive insulin; T-BIL, total bilirubin.

### Changes in CTGF level by sequential biopsy in patients with NAFLD

**Fig 5:**
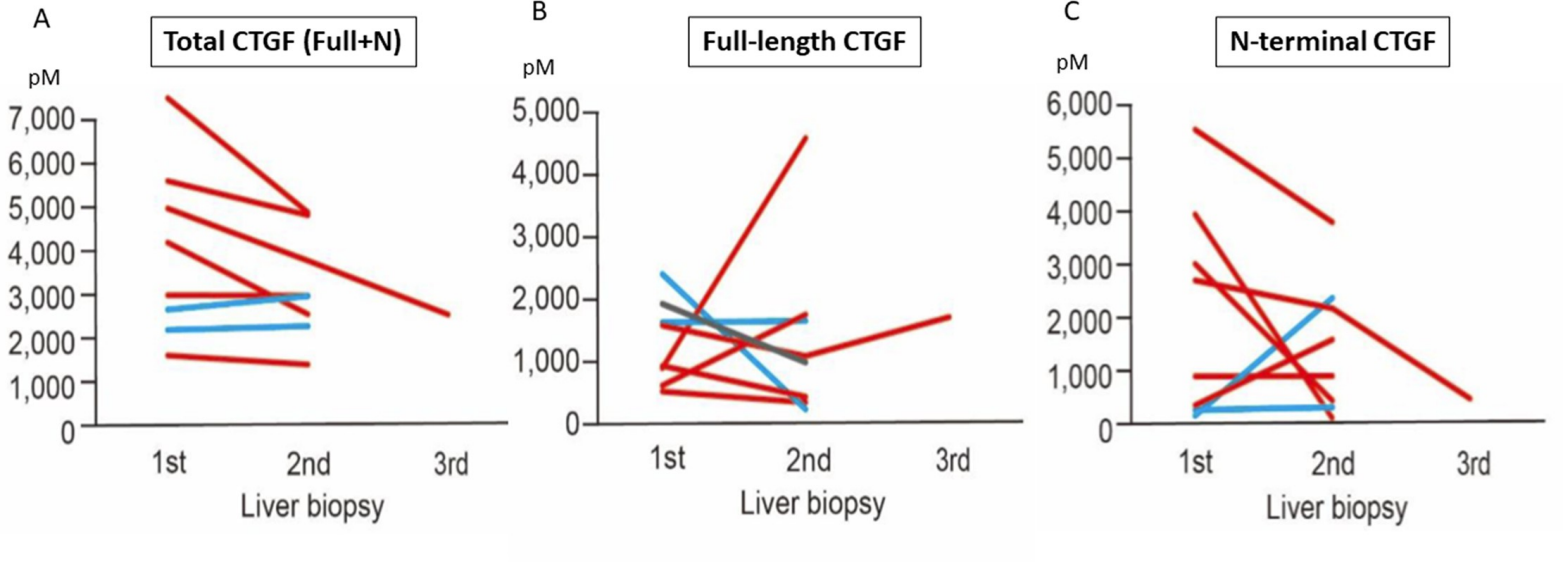
Changes in CTGF level at sequential liver biopsy in patients with NAFLD. Serum levels of **(A)** total CTGF (full-length + N-terminal fragment), **(B)** full-length CTGF, and **(C)** N-terminal CTGF fragment were determined in eight patients with NAFLD who underwent sequential liver biopsy. The blue lines indicate the patients in whom the fibrosis stage pathologically improved. The red lines indicate the patients who did not improve. All data were determined in duplicate, and the average values are shown. CTGF, connective tissue growth factor; NAFLD, nonalcoholic fatty liver disease.

Changes in the CTGF level in patients with NAFLD who underwent sequential biopsy were assessed. Because no correlation between the CTGF level and fibrosis stage was observed, we hypothesized that individual-patient variances may exist in the basal CTGF level, masking the ability to estimate the fibrosis progression according to the CTGF level. To test this hypothesis, we examined the changes in the CTGF level at different time points in eight patients with NAFLD who had a history of sequential biopsy (Table 3). In two of these patients (Patients 2 and 4), the liver fibrosis stage improved after a 29 kg reduction in body weight or reconstruction of the gastrointestinal tract. The liver enzyme levels decreased to the reference range, and the liver fibrosis stage improved from F3 to F1–2 after 6 years and from F2–3 to F2 after 2 years. The liver fibrosis stage progressed over time in the other six patients. As shown in Fig 5, although the total CTGF level (Fig 5A) decreased over time in all patients except two in whom the liver fibrosis stage improved, the serum levels of full-length CTGF (Fig 5B) and N-terminal CTGF fragment (Fig 5C) changed regardless of liver fibrosis progression or improvement. This suggests that the level of CTGF (either full-length or N-terminal fragment) was not correlated with the liver fibrosis stage in patients with NAFLD, even considering the baseline differences in the CTGF level between individual patients.

**Table 3:** CTGF level and fibrosis stage in patients who underwent paired liver biopsy.

	First biopsy		Second biopsy		Third biopsy		
Patient	Age (years)	Fibrosis stage	Age (years)	Fibrosis stage	Age (years)	Fibrosis stage	Platelet count (×10^4^/μL) at first/second/third biopsy
1	60	2	68	3–4			12.5/11.7
2*	49	3	55	1–2			15.3/19.5
3	65	0–1	70	2–3			29.7/30.5
4*	33	2–3	35	2			24.0/26.0
5	46	2–3	50	3–4			24.7/19.5
6	37	2	40	3	42	3	13.5/14.4/18.4
7	60	2–3	62	3–4			24.6/24.7
8	61	1	68	2–3			26.4/27.6

*Patients with improvement of liver fibrosis at second biopsy

CTGF, connective tissue growth factor.

## Discussion

The serum level of CTGF was correlated with the fibrosis stage in patients with FALD but not in those with NAFLD, indicating that it may be a good surrogate marker for estimating the liver fibrosis stage in patients with FALD. In fact, the plasma level of CTGF is a potential biomarker in patients with pulmonary fibrosis [25]. We found that in patients with FALD, the full-length CTGF level but not the total CTGF level was correlated with the stage of liver fibrosis. Historically, only total CTGF, which consists of full-length CTGF and the N-terminal CTGF fragment, could be measured. However, the recent development of the antibody against module 4 of full-length CTGF enabled us to specifically measure the level of full-length CTGF. By subtracting this level from the total CTGF level determined using the antibody against module 2, we could also examine the N-terminal CTGF fragment level. This enabled us to measure the two types of CTGF separately. As previously described [7, 8], full-length CTGF is released from platelets *in vitro* after blood collection, which may have contributed to the significant inverse correlation with the platelet count in patients with FALD. The platelet count is generally inversely correlated with the progression of liver fibrosis [17]; however, a clinical discrepancy is sometimes encountered. We expected that full-length CTGF would be a more reliable marker of the liver fibrosis stage in patients with FALD because the full-length CTGF level better reflected the liver fibrosis stage with a high Pearson correlation coefficient. To confirm the accuracy of noninvasive fibrosis markers for patients with FALD, we evaluated platelet count, FIB-4 index, MELD-XI, and CTGF level. In the multivariate analysis and AUC of fit to the fibrosis stage in patients with FALD, full-length CTGF was identified as the predictor of the fibrosis stage. Taken together, these findings indicate that the full-length CTGF level may be a more reliable marker for estimating the liver fibrosis stage in patients with FALD.

To further confirm that the CTGF level contributes to disease development in patients with FALD, we evaluated CVP by cardiac catheterization. Only the full-length CTGF level tended to be correlated with CVP. Inuzuka *et al*. [26] reported that CVP and atrioventricular valve regurgitation were associated with the development of fibrosis in patients with FALD. Therefore, FALD development might reflect the inverse correlation between high CVP and the full-length CTGF level. In terms of the pharmacokinetics of CTGF within the body, it has been observed that CTGF undergoes rapid internalization via low-density lipoprotein receptor-related protein 1 (LRP1) in hepatocytes, followed by subsequent intracellular degradation after endocytosis in vitro [27]. Conversely, during the active angiogenic stages, matrix metalloproteinases (MMPs) are known to be overproduced [28]. Therefore, it is possible that CTGF may undergo degradation by MMPs. Although the specific mechanism underlying this process was not identified in our study, we speculate that the decrease in full-length CTGF levels and the increase in N-terminal CTGF fragment levels may result from cleavage of CTGF during the development of fibrosis in patients with FALD. Nevertheless, further investigation is required to fully understand these mechanisms.

In contrast to patients with FALD cases, the CTGF level was not correlated with the liver fibrosis stage in patients with NAFLD. Although we considered the possibility of individual-patient differences in the CTGF level, there was clearly no correlation between the CTGF level and liver fibrosis stage in patients with NAFLD who underwent sequential biopsy. This may be reflected in the pathogenetic differences in liver fibrosis between NAFLD and FALD. Full-length CTGF is mainly produced by endothelial cells *in vivo* [29]. It is highly possible that activation of endothelial cells, such as liver sinusoidal endothelial cells, may be closely involved in FALD-related liver fibrosis [30] but not in NAFLD-related liver fibrosis. Although several studies have revealed the crucial role of CTGF in liver fibrosis [5, 31], etiological differences should be considered. The usefulness of the CTGF level as a marker of liver fibrosis remains to be determined in other liver diseases, such as viral hepatitis and alcoholic liver diseases. FALD is a representative complication in patients long after the Fontan operation. Because some patients develop advanced liver disease in the long term, noninvasive markers to estimate liver fibrosis have been explored [15]. The full-length CTGF level may be promising marker; however, this study had two main limitations. First, it was a single-center study, and pathological examination could not be performed in all cases. Further prospective studies are necessary to determine the usefulness of CTGF measurement for estimating the fibrosis stage in patients with FALD. Second, an additional investigation is needed to elucidate the mechanism of proteolytic processing of CTGF by progression of fibrosis.

In conclusion, a new detection antibody targeting the C-terminal of CTGF enabled measurement of the serum levels of the two forms of CTGF. The full-length CTGF level may better reflect the liver fibrosis stage than other markers in patients with FALD cases and is inversely correlated with increases in CVP.

## Supporting information

S1 Fig The accuracy of predicting the fibrosis stage was determined by receiver operating characteristic curve analysis in patients with FALD. The AUC for the platelet count, FIB-4 index, and full-length CTGF was 0.744, 0.567, and 0.822, respectively. Full-length CTGF was the best predictor of fibrosis in patients with FALD. AUC, area under the curve; CTGF, connective tissue growth factor; FALD, Fontan-associated liver disease; FIB-4, fibrosis-4.
(TIF)

## References

[1] Kono M, Nakamura Y, Suda T, Kato M, Kaida Y, Hashimoto D, et al. Plasma CCN2 (connective tissue growth factor; CTGF) is a potential biomarker in idiopathic pulmonary fibrosis (IPF). Clin Chim Acta. 2011;412(23–24):2211–5. Epub 20110812. doi: 10.1016/j.cca.2011.08.008 . 21864521

[2] di Mola FF, Di Sebastiano P, Gardini A, Innocenti P, Zimmermann A, Büchler MW, et al. Differential expression of connective tissue growth factor in inflammatory bowel disease. Digestion. 2004;69(4):245–53. Epub 20040714. doi: 10.1159/000079845 . 15256831

[3] Valentijn FA, Knoppert SN, Pissas G, Rodrigues-Diez RR, Marquez-Exposito L, Broekhuizen R, et al. CCN2 Aggravates the Immediate Oxidative Stress-DNA Damage Response following Renal Ischemia-Reperfusion Injury. Antioxidants (Basel). 2021;10(12). Epub 20211220. doi: 10.3390/antiox10122020 ; PubMed Central PMCID: PMC8698829. 34943123 PMC8698829

[4] Hora C, Negro F, Leandro G, Oneta CM, Rubbia-Brandt L, Muellhaupt B, et al. Connective tissue growth factor, steatosis and fibrosis in patients with chronic hepatitis C. Liver Int. 2008;28(3):370–6. Epub 20071101. doi: 10.1111/j.1478-3231.2007.01608.x . 17976159

[5] Gressner OA, Gressner AM. Connective tissue growth factor: a fibrogenic master switch in fibrotic liver diseases. Liver Int. 2008;28(8):1065–79. doi: 10.1111/j.1478-3231.2008.01826.x . 18783549

[6] Brigstock DR. Connective tissue growth factor (CCN2, CTGF) and organ fibrosis: lessons from transgenic animals. J Cell Commun Signal. 2010;4(1):1–4. Epub 20091002. doi: 10.1007/s12079-009-0071-5 ; PubMed Central PMCID: PMC2821473. 19798591 PMC2821473

[7] Cicha I, Garlichs CD, Daniel WG, Goppelt-Struebe M. Activated human platelets release connective tissue growth factor. Thromb Haemost. 2004;91(4):755–60. doi: 10.1160/TH03-09-0602 . 15045137

[8] Kubota S, Kawata K, Yanagita T, Doi H, Kitoh T, Takigawa M. Abundant retention and release of connective tissue growth factor (CTGF/CCN2) by platelets. J Biochem. 2004;136(3):279–82. doi: 10.1093/jb/mvh126 . 15598883

[9] Zhang D, Wang NY, Yang CB, Fang GX, Liu W, Wen J, et al. The clinical value of serum connective tissue growth factor in the assessment of liver fibrosis. Dig Dis Sci. 2010;55(3):767–74. Epub 20090318. doi: 10.1007/s10620-009-0781-9 . 19294506

[10] Colak Y, Senates E, Coskunpinar E, Oltulu YM, Zemheri E, Ozturk O, et al. Concentrations of connective tissue growth factor in patients with nonalcoholic fatty liver disease: association with liver fibrosis. Dis Markers. 2012;33(2):77–83. doi: 10.3233/DMA-2012-0907 ; PubMed Central PMCID: PMC3810787. 22846210 PMC3810787

[11] Miyazaki O, Kurashita S, Fukamachi I, Endo K, Ng PS, Takehara K. Subtraction method for determination of N-terminal connective tissue growth factor. Ann Clin Biochem. 2010;47(Pt 3):205–11. Epub 20100414. doi: 10.1258/acb.2010.009182 . 20392752

[12] Hagström H, Nasr P, Ekstedt M, Hammar U, Stål P, Hultcrantz R, et al. Fibrosis stage but not NASH predicts mortality and time to development of severe liver disease in biopsy-proven NAFLD. J Hepatol. 2017;67(6):1265–73. Epub 20170810. doi: 10.1016/j.jhep.2017.07.027 . 28803953

[13] Boursier J, Hagström H, Ekstedt M, Moreau C, Bonacci M, Cure S, et al. Non-invasive tests accurately stratify patients with NAFLD based on their risk of liver-related events. J Hepatol. 2022;76(5):1013–20. Epub 20220119. doi: 10.1016/j.jhep.2021.12.031 . 35063601

[14] Kogiso T, Tokushige K. Fontan-associated liver disease and hepatocellular carcinoma in adults. Sci Rep. 2020;10(1):21742. Epub 20201210. doi: 10.1038/s41598-020-78840-y ; PubMed Central PMCID: PMC7728791. 33303924 PMC7728791

[15] Emamaullee J, Khan S, Weaver C, Goldbeck C, Yanni G, Kohli R, et al. Non-invasive biomarkers of Fontan-associated liver disease. JHEP Rep. 2021;3(6):100362. Epub 20210914. doi: 10.1016/j.jhepr.2021.100362 ; PubMed Central PMCID: PMC8517550. 34693238 PMC8517550

[16] Kim BK, Tamaki N, Imajo K, Yoneda M, Sutter N, Jung J, et al. Head to head comparison between MEFIB, MAST, and FAST for detecting stage 2 fibrosis or higher among patients with NAFLD. J Hepatol. 2022. Epub 20220813. doi: 10.1016/j.jhep.2022.07.020 . 35973577

[17] Isoura Y, Yamamoto A, Cho Y, Ehara E, Jogo A, Suzuki T, et al. Platelet count and abdominal dynamic CT are useful in predicting and screening for gastroesophageal varices after Fontan surgery. PLoS One. 2021;16(10):e0257441. Epub 20211007. doi: 10.1371/journal.pone.0257441 ; PubMed Central PMCID: PMC8496823. 34618830 PMC8496823

[18] Kogiso T, Sagawa T, Taniai M, Shimada E, Inai K, Shinohara T, et al. Risk factors for Fontan-associated hepatocellular carcinoma. PLoS One. 2022;17(6):e0270230. Epub 20220617. doi: 10.1371/journal.pone.0270230 ; PubMed Central PMCID: PMC9205474. 35714161 PMC9205474

[19] Tokushige K, Ikejima K, Ono M, Eguchi Y, Kamada Y, Itoh Y, et al. Evidence-based clinical practice guidelines for nonalcoholic fatty liver disease/nonalcoholic steatohepatitis 2020. J Gastroenterol. 2021. Epub 20210917. doi: 10.1007/s00535-021-01796-x . 34533632 PMC8531062

[20] Tokushige K, Ikejima K, Ono M, Eguchi Y, Kamada Y, Itoh Y, et al. Evidence-based clinical practice guidelines for nonalcoholic fatty liver disease/nonalcoholic steatohepatitis 2020. Hepatol Res. 2021;51(10):1013–25. Epub 20210917. doi: 10.1111/hepr.13688 . 34533266

[21] Castera L. Non-invasive tests for liver fibrosis in NAFLD: Creating pathways between primary healthcare and liver clinics. Liver Int. 2020;40 Suppl 1:77–81. doi: 10.1111/liv.14347 . 32077617

[22] Matthews DR, Hosker JP, Rudenski AS, Naylor BA, Treacher DF, Turner RC. Homeostasis model assessment: insulin resistance and beta-cell function from fasting plasma glucose and insulin concentrations in man. Diabetologia. 1985;28(7):412–9. doi: 10.1007/BF00280883 . 3899825

[23] Evans WN, Acherman RJ, Ciccolo ML, Carrillo SA, Galindo A, Rothman A, et al. MELD-XI Scores Correlate with Post-Fontan Hepatic Biopsy Fibrosis Scores. Pediatr Cardiol. 2016;37(7):1274–7. Epub 2016/06/14. doi: 10.1007/s00246-016-1428-1 . 27300556

[24] Brunt EM, Janney CG, Di Bisceglie AM, Neuschwander-Tetri BA, Bacon BR. Nonalcoholic steatohepatitis: a proposal for grading and staging the histological lesions. Am J Gastroenterol. 1999;94(9):2467–74. doi: 10.1111/j.1572-0241.1999.01377.x . 10484010

[25] Effendi WI, Nagano T. Connective Tissue Growth Factor in Idiopathic Pulmonary Fibrosis: Breaking the Bridge. Int J Mol Sci. 2022;23(11). Epub 20220528. doi: 10.3390/ijms23116064 ; PubMed Central PMCID: PMC9181498. 35682743 PMC9181498

[26] Inuzuka R, Nii M, Inai K, Shimada E, Shinohara T, Kogiso T, et al. Predictors of liver cirrhosis and hepatocellular carcinoma among perioperative survivors of the Fontan operation. Heart. 2023;109(4):276–82. Epub 20230127. doi: 10.1136/heartjnl-2022-320940 . 35768191

[27] Segarini PR, Nesbitt JE, Li D, Hays LG, Yates JR, Carmichael DF. The low density lipoprotein receptor-related protein/alpha2-macroglobulin receptor is a receptor for connective tissue growth factor. J Biol Chem. 2001;276(44):40659–67. Epub 20010822. doi: 10.1074/jbc.M105180200 . 11518710

[28] Sang QX. Complex role of matrix metalloproteinases in angiogenesis. Cell Res. 1998;8(3):171–7. doi: 10.1038/cr.1998.17 . 9791730

[29] Bradham DM, Igarashi A, Potter RL, Grotendorst GR. Connective tissue growth factor: a cysteine-rich mitogen secreted by human vascular endothelial cells is related to the SRC-induced immediate early gene product CEF-10. J Cell Biol. 1991;114(6):1285–94. doi: 10.1083/jcb.114.6.1285 ; PubMed Central PMCID: PMC2289134. 1654338 PMC2289134

[30] Kawai H, Osawa Y, Matsuda M, Tsunoda T, Yanagida K, Hishikawa D, et al. Sphingosine-1-phosphate promotes tumor development and liver fibrosis in mouse model of congestive hepatopathy. Hepatology. 2022;76(1):112–25. Epub 20211222. doi: 10.1002/hep.32256 . 34855990

[31] Hao C, Xie Y, Peng M, Ma L, Zhou Y, Zhang Y, et al. Inhibition of connective tissue growth factor suppresses hepatic stellate cell activation in vitro and prevents liver fibrosis in vivo. Clin Exp Med. 2014;14(2):141–50. Epub 20130302. doi: 10.1007/s10238-013-0229-6 . 23456538

