# Supplementary figures and images for: Serum level of full-length connective tissue growth factor reflects liver fibrosis stage in patients with Fontan-associated liver disease
Source: PLoS One. 2024 Jan 2;19(1):e0296375. doi: 10.1371/journal.pone.0296375 (PMC10760884; doi:10.1371/journal.pone.0296375)

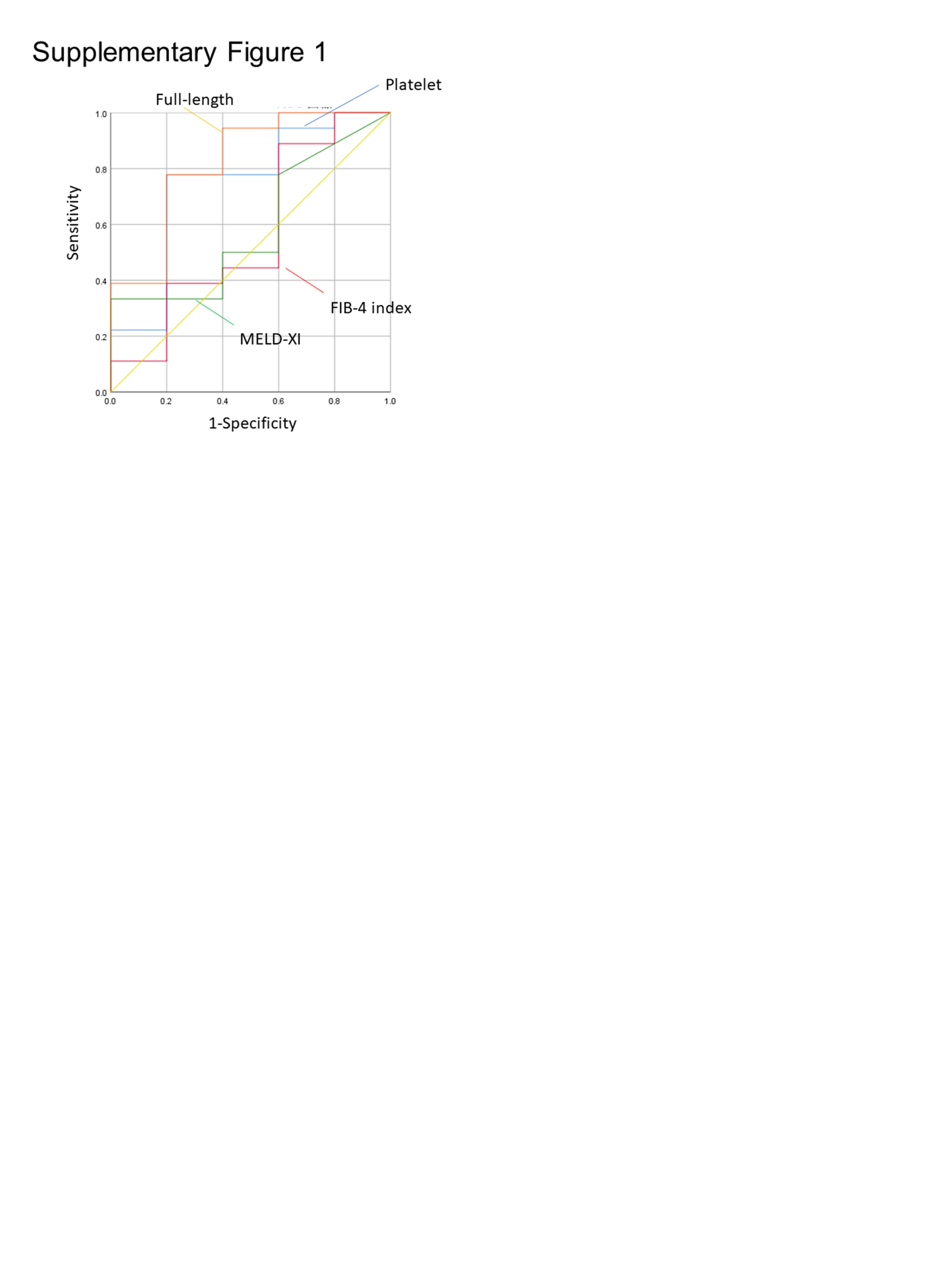

Supplement: S1 Fig — The AUC for the platelet count, FIB-4 index, and full-length CTGF was 0.744, 0.567, and 0.822, respectively. Full-length CTGF was the best predictor of fibrosis in patients with FALD. AUC, area under the curve; CTGF, connective tissue growth factor; FALD, Fontan-associated liver disease; FIB-4, fibrosis-4. (TIF) [file pone.0296375.s001.TIF]
